# Valacyclovir for the prevention of cytomegalovirus infection after kidney transplantation

**DOI:** 10.1186/s12879-025-10671-6

**Published:** 2025-03-05

**Authors:** Jin Sug Kim, Na Rae Lee, Kyun-Ik Park, Hyeon Seok Hwang, Sang Ho Lee, Byung Ha Chung, Cheol Woong Jung, Jang-Hee Cho, Woo Yeong Park, Hyo Jin Kim, Jong Cheol Jeong, Jaeseok Yang, Yu Ho Lee, Jae Berm Park, Jin Seok Jeon, Juhan Lee, Yeong Hoon Kim, Soo Jin Na Choi, Jieun Oh, Hye Eun Yoon, Deok Gie Kim, Ho Sik Shin, Tae Hyun Ban, Myoung Soo Kim, Min Jung Ko, Kyung Hwan Jeong, KOTRY study group

**Affiliations:** 1 Division of Nephrology, Department of Internal Medicine, Kyung Hee University College of Medicine, Kyung Hee University Medical Center, 26, Kyungheedae-Ro, Dongdaemun-Gu, Seoul, 02447 Republic of Korea; 2 Division of Healthcare Technology Assessment Research, National Evidence-Based Healthcare Collaborating Agency, 400, Neungdong-Ro, Gwangjin-Gu, Seoul, 04933 Republic of Korea; 3 Division of Nephrology, Department of Internal Medicine, Kyung Hee University College of Medicine, Kyung Hee University Hospital at Gangdong, Seoul, Republic of Korea; 4 Division of Nephrology, Department of Internal Medicine, Seoul St. Mary’S Hospital, Seoul, Republic of Korea; 5 Department of Surgery, Korea University Anam Hospital, Seoul, Republic of Korea; 6 Department of Internal Medicine, School of Medicine, Kyungpook National University Hospital, Daegu, Republic of Korea; 7 Division of Nephrology, Department of Internal Medicine, Keimyung University School of Medicine, Keimyung University Dongsan Hospital, Daegu, Republic of Korea; 8 Division of Nephrology, Department of Internal Medicine, Pusan National University Hospital, Pusan National University School of Medicine, Busan, Republic of Korea; 9 Division of Nephrology, Seoul National University Bundang Hospital, Seongnam, Republic of Korea; 10 Division of Nephrology, Department of Internal Medicine, Yonsei University College of Medicine, Severance Hospital, Seoul, Republic of Korea; 11 Division of Nephrology, Department of Internal Medicine, CHA Bundang Medical Center, CHA University, Seongnam, Republic of Korea; 12 Department of Surgery, Samsung Medical Center, Sungkyunkwan University School of Medicine, Seoul, Republic of Korea; 13 Department of Internal Medicine, Soonchunhyang University Seoul Hospital, Seoul, Republic of Korea; 14 Department of Surgery, Yonsei University College of Medicine, Seoul, Republic of Korea; 15 Department of Internal Medicine, Inje University Busan Paik Hospital, Busan, Republic of Korea; 16 Department of Surgery, Chonnam National University Medical School, Gwangju, Republic of Korea; 17 Department of Internal Medicine, Kangdong Sacred Heart Hospital, Hallym University College of Medicine, Seoul, Republic of Korea; 18 Division of Nephrology, Department of Internal Medicine, Incheon St. Mary’s Hospital, College of Medicine, The Catholic University of Korea, Incheon, Republic of Korea; 19 Department of Surgery, Yonsei University Wonju College of Medicine, Wonju Severance Christian Hospital, Wonju, Republic of Korea; 20 Renal Division, Department of Internal Medicine, Gospel Hospital, Kosin University College of Medicine, Busan, South Korea; 21 Transplantation Research Institute, Kosin University College of Medicine, Busan, South Korea; 22 Division of Nephrology, Department of Internal Medicine, Eunpyeong St. Mary’s Hospital, College of Medicine, The Catholic University of Korea, Seoul, Republic of Korea

**Keywords:** Cytomegalovirus, Kidney transplantation, Valacyclovir

## Abstract

**Background:**

Cytomegalovirus (CMV) infection is a frequent complication after kidney transplantation (KT) and has various effects on recipient and graft survival. Although guidelines recommend anti-viral prophylaxis with ganciclovir or valganciclovir, there is a demand for alternative regimen for CMV prevention. We investigated the effects of a 3-month valacyclovir-based prophylaxis on CMV infection and clinical outcomes in KT recipients using a nationwide cohort.

**Methods:**

Overall, 2,584 KT recipients from 20 transplant centers registered with the Korean Organ Transplantation Registry between May 2014 and December 2019 were analyzed in this study. The recipients were divided into valacyclovir prophylaxis and non-prophylaxis groups, a 1:3 propensity score matching was performed, and 1,036 recipients (291 and 745 in the prophylaxis and non-prophylaxis groups, respectively) were analyzed. The impact of valacyclovir-based prophylaxis on CMV after KT, other clinical outcomes, and the risk factors for CMV infection development were investigated.

**Results:**

The prophylaxis group showed a lower incidence of CMV infection and rejection compared to the non-prophylaxis group (3.64 vs. 10.25 events/100 person-years and 1.85 vs. 7.27 events/100 person-years, respectively). Valacyclovir prophylaxis, donor age, deceased donor, length of hospitalization after KT, anti-thymocyte globulin use, and CMV serological mismatch between the donor and recipient were independent risk factors for CMV infection after KT.

**Conclusions:**

Valacyclovir prophylaxis after KT significantly reduced CMV infection and rejection. We suggest that valacyclovir could be considered as an alternative strategy for CMV prophylaxis after KT. However, our study has limitations, including its retrospective design, variability in valacyclovir dosing and CMV monitoring, and unassessed confounding factors. Further prospective studies with standardized protocols and larger cohorts are needed to validate our findings.

**Supplementary Information:**

The online version contains supplementary material available at 10.1186/s12879-025-10671-6.

## Background

Cytomegalovirus (CMV) infection is a frequent infectious complication after kidney transplantation (KT) and is associated with significant morbidity and mortality [1, 2]. Prior studies have reported that 8–60% of KT recipients experience CMV infection and disease, with the main onset occurring 6 weeks to 6 months after KT [3–5]. Risk factors for CMV infection after KT include CMV serological mismatch between donor and recipient, use of anti-T cell antibody therapy, age and comorbidities of the recipient, other concurrent infections, and presence of graft rejection [6, 7]. CMV serological mismatch between the donor and recipient is reported to have a significant influence on CMV infection and disease after organ transplantation. The risk of CMV infection associated with the serological status of the donor and recipient are classified as follows: donor(D) + /recipient(R) − , high risk; D + /R + or D − /R + , intermediate risk; and D − /R − , low risk [8, 9].

CMV increases risk of the rejection, graft loss, opportunistic superinfections, post-transplant diabetes mellitus, cardiac complications, and mortality in KT recipients [10–15]. Considering the poor clinical impact of CMV on KT recipients, current international guidelines recommend anti-viral prophylaxis for CMV infection in these populations. The guidelines recommend that KT recipients with intermediate or high risk for CMV infection receive prophylaxis with ganciclovir or valganciclovir for 3–6 months after KT [16–18]. Although intravenous ganciclovir is considered the initial drug of choice for CMV prophylaxis, it is inconvenient for patients to be hospitalized or visit the hospital frequently for drug use, and there are concerns about side effects such as neutropenia and nephrotoxicity. Valganciclovir is currently the preferred drug for CMV prophylaxis because it can be used orally and has a high bioavailability [5, 19]. However, it cannot be used in all KT recipients requiring CMV prophylaxis because of limitations, such as high cost and side effects. In Korea, valganciclovir for CMV prophylaxis is not reimbursed by the national health insurance system for patients other than those categorized as high risk. This limitation necessitates the exploration and utilization of alternative agents by clinicians to effectively manage CMV prophylaxis in KT recipients.

Previous studies have demonstrated the clinical efficacy and tolerability of valacyclovir for CMV prophylaxis. Lowance et al. [20] reported that valacyclovir-based CMV prophylaxis reduced CMV disease, acute graft rejection, and other infectious complications in KT recipients, with minimal adverse effects. Other researchers showed that oral valacyclovir is not inferior to oral valganciclovir or oral ganciclovir in terms of CMV prevention and safety [21, 22]. Based on previous studies, we assumed that oral valacyclovir could be an alternative strategy for CMV prophylaxis in KT recipients. In this study, we investigated the efficacy of oral valacyclovir-based prophylaxis for three months to prevent CMV infection after KT using a nationwide cohort. We also analyzed the impact of valacyclovir-based CMV prophylaxis on clinical outcomes, including cardiac events, rejection, graft loss, renal dysfunction, and all-cause mortality, and investigated the risk factors for the development of CMV infection after KT.

## Methods

### Study population and design

The study population was obtained from the Korean Organ Transplantation Registry (KOTRY) database, which is a prospective, multicenter nationwide Korean transplantation cohort. The KOTRY database includes information on five types of solid organ transplantation in Korea: kidney, liver, pancreas, heart, and lung. Further details of the cohort design have been previously described [23, 24]. This study included 3,214 kidney recipients from 20 transplant centers registered in the KOTRY database between May 2014 and December 2019. The KOTRY database prospectively collects comprehensive data, including epidemiological trends, graft-related outcomes, and mortality. For this analysis, we utilized data collected up to December 2020.

**Fig. 1:**
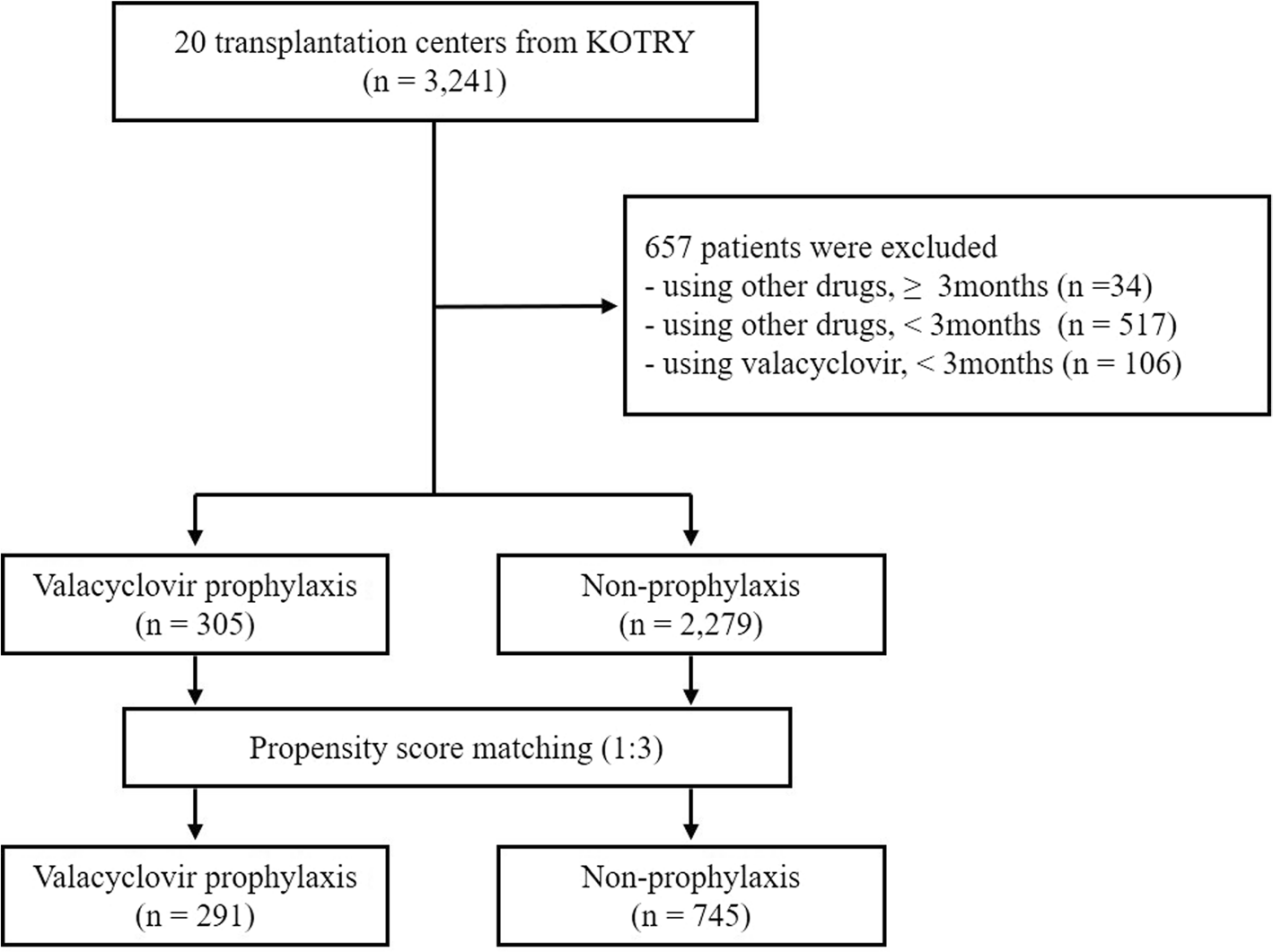
Study flowchart. KOTRY; Korean Organ Transplantation Registry

As shown in Fig. 1, recipients who used anti-viral agents other than valacyclovir for CMV prophylaxis or those with a CMV prophylaxis duration of less than 3 months were excluded. A total of 2,584 KT recipients were included in the analysis and categorized into two groups: the valacyclovir prophylaxis group and the non-prophylaxis group. The valacyclovir prophylaxis group commenced valacyclovir treatment during the pre-discharge period following KT, while the non-prophylaxis group did not receive any antiviral agents for prophylactic purposes. We conducted a propensity score-matched analysis, with the methodology and relevant details outlined in the subsequent statistical analysis section.

All the study procedures complied with the principles of the Declaration of Helsinki. The study protocol was reviewed and approved by the Institutional Review Board of each center and the National Evidence-based Healthcare Collaborating Agency (NECA-IRB number: NECAIRB21-002). The need for informed consent was waived by the Institutional Review Board of each center and the National Evidence-based Healthcare Collaborating Agency.

### Variables and study outcomes

Demographic, clinical, and laboratory data were collected for each recipient. KT-related data including donor age and sex, whether the donor was deceased or not, ABO incompatibility, presence of donor-specific antibody (DSA), types of immunosuppressive drugs, and CMV serological mismatch between the donor and the recipient (CMV seropositive donor and CMV seronegative recipient) were also recorded. Due to the limitations of the cohort design, the KOTRY database lacks information on CMV prophylaxis, the occurrence of CMV infection, or disease. Therefore, we retrospectively collected information on whether prophylaxis was provided, duration of prophylaxis, type of anti-CMV drug used for prophylaxis, and the occurrence of CMV infection or disease within one year after KT. Each transplant center was provided with a standardized survey form for data collection. Data collection at each center was conducted with cross-verification by at least two experienced researchers to ensure accuracy and consistency.

The primary outcomes of this study were the occurrence of CMV infection and disease within one year of KT. CMV infection and disease were defined based on the recently published guidelines [17, 25]. CMV infection was defined as the isolation of the virus or detection of antigens or nucleic acids in any body fluid or tissue specimen. CMV disease was defined as CMV infection with accompanying symptoms and was classified according to the affected organ: pneumonia, gastrointestinal disease, hepatitis, retinitis, encephalitis and ventriculitis, nephritis, cystitis, myocarditis, pancreatitis, other end-organ disease categories, and CMV syndrome. All 20 transplant centers involved in the study monitored CMV by detecting DNAemia. CMV DNAemia was defined as a positive result from quantitative CMV real-time polymerase chain reaction (PCR) testing of whole blood samples. The monitoring schedule for CMV DNAemia was determined according to the protocol established by each participating center. The secondary outcomes were the clinical outcomes after KT in terms of cardiac events, rejection, graft loss, renal dysfunction, and all-cause mortality. These outcomes were monitored from the date of patient enrollment until December 2020. Cardiac events included myocardial infarction, ischemic heart disease, new-onset congestive heart failure, and cardiac death. Rejection included both clinical and biopsy-proven rejection, with biopsy-proven rejection encompassing both Acute cellular and antibody-mediated rejection. Graft loss was defined as dependence on dialysis for > 3 months. Renal dysfunction was defined as > 50% increase in creatinine or a > 30% decrease in the estimated glomerular filtration rate (eGFR) compared to measurements at discharge. The eGFR was calculated using the Chronic Kidney Disease Epidemiology Collaboration (CKD-EPI) equation [26]. All-cause mortality included both cardiac and non-cardiac deaths.

### Statistical analysis

Continuous variables are expressed as mean ± standard deviations (SDs), and categorical data are presented as frequencies and percentages. Data were analyzed using Student's t-test, chi-squared test, or Fisher’s exact test, as appropriate. Survival curves were estimated using the Kaplan–Meier method and compared using the log-rank test. Univariate and multivariate Cox regression analyses were used to determine the association of variables with CMV infection. The results are presented as hazard ratios (HRs) ± 95% confidence intervals (CI).

To balance the differences between the two groups and reduce potential selection bias, a propensity score was estimated using a multivariable logistic regression model. Variables included in the logistic regression model used to deduce the propensity score were age and sex of the recipient; body mass index; cause of end stage renal disease (ESRD); history of diabetes mellitus, hypertension, and cardiovascular disease; eGFR; serum levels of albumin and hemoglobin at baseline; re-transplantation; age and sex of the donor; desensitization; type of induction and maintenance immunosuppressive drugs; ABO incompatibility; presence of DSA; length of hospitalization after KT; and CMV serological mismatch between the donor and recipient. The prophylaxis and non-prophylaxis groups were subsequently matched using a 1:3 matching algorithm for propensity scores. All statistical analyses were performed using the SAS software, version 9.3 (SAS Institute Inc., Cary, NC, USA). All reported p-values were 2-sided, and p-values less than 0.05 were considered statistically significant.

## Results

### Baseline characteristics of the study population

The clinical characteristics, laboratory findings, KT-related data, and medications used are presented in Table 1. Among the 2,584 KT recipients, 305 (11.8%) received valacyclovir prophylaxis. There were no significant differences in age, sex, or body mass index between the two groups. The baseline hemoglobin and creatinine levels at discharge showed significant differences between the two groups. The prophylaxis group showed a higher frequency of re-transplantation (9.2% vs. 5.6%, *p* = 0.01) and the presence of DSA (8.6% vs. 5.6%, *p* < 0.01). The prophylaxis group used more intravenous immunoglobulin (IVIG) (7.5% vs. 4.9%, p < 0.01) and anti-thymocyte globulin (ATG) (20.7% vs. 8.6%, *p* < 0.01) compared to the non-prophylaxis group. Recipients at high risk for CMV infection, as assessed by CMV seropositivity (CMV serological status: D + /R −), were more frequently observed in the prophylaxis group (3.3% vs. 0.6%, *p* < 0.01). In contrast, there were no significant differences between the two groups in the frequency of recipients at moderate or low risk for CMV infection. After 1:3 propensity score-matching, 1,036 recipients (291 and 745 in the prophylaxis and non-prophylaxis groups, respectively) were matched. In the propensity score-matched populations, no significant differences were observed between the two groups with respect to clinical characteristics, laboratory findings, KT-related data, and medications.

**Table 1:** Baseline characteristics of the study population

	Before matching				After matching			
	Prophylaxis (*n* = 305)	Non-prophylaxis (*n* = 2279)	*p*	STD	Prophylaxis (*n* = 291)	Non-prophylaxis (*n* = 745)	*p*	STD
Age (years)	48.85 ± 11.10	49.34 ± 11.75	0.49	−0.04	48.85 ± 11.21	48.90 ± 12.03	0.95	0.00
Male sex (n, %)	177 (58.0)	1398 (61.3)	0.27	−0.07	167 (57.4)	425 (57.1)	0.92	0.01
Body mass index (kg/m^2^)	22.77 ± 3.27	23.12 ± 3.55	0.11	−0.10	22.82 ± 3.28	22.80 ± 3.33	0.91	0.01
Cause of ESRD (n, %)			< 0.01				0.89	
Diabetes mellitus	90 (29.5)	510 (22.38)		0.16	86 (29.6)	221 (29.7)		0
Hypertension	26 (8.5)	402 (17.6)		−0.27	25 (8.6)	72 (9.7)		−0.04
Glomerulonephritis	141 (46.2)	688 (30.2)		0.33	134 (46.1)	321 (43.1)		0.06
ADPKD	9 (2.9)	120 (5.3)		−0.12	9 (3.1)	31 (4.2)		−0.06
Others	14 (4.6)	73 (3.2)		0.07	13 (4.5)	30 (4.0)		0.02
Unknown	25 (8.2)	486 (21.3)		−0.38	24 (8.3)	70 (9.4)		−0.04
Duration of dialysis (months)	50.18 ± 66.78	44.43 ± 59.84	0.15	0.09	49.64 ± 66.87	47.92 ± 63.17	0.7	0.03
Diabetes mellitus (n, %)	104 (34.1)	689 (30.23)	0.17	0.08	100 (34.4)	257 (34.5)	0.97	0.00
Hypertension (n, %)	267 (87.5)	2048 (89.9)	0.42	−0.07	257 (88.3)	664 (89.1)	0.71	−0.03
Cardiovascular disease (n, %)	32 (10.5)	258 (11.3)	0.23	−0.03	31 (10.7)	78 (10.5)	0.78	0.01
Baseline albumin (g/dL)	4.1 ± 0.57	4.11 ± 0.60	0.90	−0.02	4.09 ± 0.57	4.07 ± 0.60	0.67	0.03
Baseline Hemoglobin (g/dL)	10.63 ± 1.48	10.85 ± 1.55	0.02	−0.15	10.63 ± 1.50	10.69 ± 1.62	0.57	−0.04
Baseline creatinine (mg/dL)	8.86 ± 2.92	8.77 ± 3.43	0.65	0.03	8.85 ± 2.93	8.77 ± 3.34	0.73	0.03
Baseline eGFR (ml/min/1.73m^2^)	6.63 ± 3.45	6.95 ± 3.97	0.18	−0.09	6.61 ± 3.41	6.76 ± 3.13	0.52	−0.05
Discharge creatinine (mg/dL)	1.1 ± 0.32	1.24 ± 0.81	< 0.01	−0.23	1.10 ± 0.33	1.11 ± 0.42	0.72	−0.03
Discharge eGFR (ml/min/1.73m^2^)	74.36 ± 17.35	70.85 ± 20.75	< 0.01	0.18	73.99 ± 17.47	74.03 ± 18.22	0.98	0.00
Re-transplantation (n, %)	28 (9.2)	127 (5.6)	0.01	0.14	24 (8.3)	56 (7.5)	0.69	0.03
Donor age (years)	47.08 ± 12.89	46.97 ± 12.71	0.89	0.01	47.30 ± 12.92	46.49 ± 13.02	0.37	0.06
Donor male sex (n, %)	162 (53.1)	1158 (50.8)	0.71	0.05	154 (52.9)	394 (52.9)	0.99	0
Deceased donor (n, %)	105 (34.4)	763 (33.5)	0.83	0.02	98 (33.7)	243 (32.6)	0.74	0.02
Desensitization (n, %)	68 (22.3)	451 (19.8)	0.31	0.06	64 (22.0)	144 (19.3)	0.34	0.07
IVIG (n, %)	23 (7.5)	112 (4.9)	< 0.01	0.11	22 (7.6)	45 (6.0)	0.67	0.06
Plasmapheresis (n, %)	29 (9.5)	211 (9.3)	0.08	0.01	28 (9.6)	58 (7.8)	0.34	0.06
Rituximab (n, %)	29 (9.5)	257 (11.3)	0.63	−0.06	28 (9.6)	60 (8.1)	0.67	0.06
ABO incompatibility (n, %)	27 (8.9)	216 (9.5)	0.73	−0.02	26 (8.9)	58 (7.8)	0.54	0.04
Presence of DSA (n, %)	17 (5.6)	197 (8.6)	< 0.01	−0.12	17 (5.8)	42 (5.6)	0.67	0.01
Number of HLA mismatching (n, %)			0.96				0.97	
0	34 (11.2)	278 (12.2)		−0.03	30 (10.3)	80 (10.7)		−0.01
1	59 (19.3)	411 (18.0)		0.03	57 (19.6)	142 (19.1)		0.01
2	48 (15.7)	409 (17.9)		−0.06	47 (16.2)	127 (17.1)		−0.02
3	72 (23.6)	521 (22.9)		0.02	70 (24.1)	188 (25.2)		−0.03
4	72 (23.6)	521 (22.9)		0.02	41 (14.1)	103 (13.8)		0.01
5	14 (4.6)	107 (4.7)		−0.01	14 (4.8)	39 (5.2)		−0.02
6	35 (11.5)	245 (10.8)		0.02	32 (11.0)	66 (8.9)		0.07
Length of hospitalization after KT (days)	16.65 ± 7.82	17.98 ± 10.77	< 0.01	−0.14	16.57 ± 7.85	16.55 ± 7.49	0.97	0
Induction immunosuppressant (n, %)			< 0.01				0.16	
Basiliximab	240 (78.7)	1977 (86.7)		−0.21	232 (79.7)	631 (84.7)		−0.13
ATG	63 (20.7)	195 (8.6)		0.35	57 (19.6)	110 (14.8)		0.13
Calcineurin inhibitors (n, %)							0.96	0.01
Tacrolimus	303 (99.3)	2246 (98.6)		0.08	289 (99.3)	741 (99.5)		−0.02
Cyclosporine	1 (0.33)	11 (0.5)		−0.02	1 (0.3)	2 (0.3)		0.01
Mycophenolate (n, %)	293 (96.1)	2009 (88.2)	< 0.01	0.30	280 (96.2)	710 (95.3)		0.05
Dosage (mg/day)	1045.33 ± 368.77	1060.89 ± 389.54	0.25	0.03	1044.59 ± 368.98	1057.84 ± 370.23	0.37	0.03
Steroid (n, %)	298 (97.7)	2242 (98.4)	0.54	−0.05	287 (98.6)	739 (99.2)	0.4	0.05
Donor CMV IgG + and Recipient CMV IgG − (n, %)	10 (3.3)	14 (0.6)	< 0.01	0.19	5 (1.7)	8 (1.1)	0.41	0.06
Donor CMV IgG + or − , and Recipient CMV IgG + (n, %)	289 (93.1)	2183 (95.8)	0.38	0.06	282 (96.9)	724 (97.2)	0.72	0.03
Donor CMV IgG – and Recipient CMV IgG – (n, %)	4 (1.3)	45(1.9)	0.41	0.05	4 (1.4)	13 (1.7)	0.57	0.04

*CMV* cytomegalovirus, *ESRD* end-stage renal disease, *ADPKD* autosomal dominant polycystic kidney disease, *STD* standardized difference, *eGFR* estimated glomerular filtration rate, *IVIG* intravenous immunoglobulin, *DSA* donor specific antibody, *HLA* human leukocyte antigens, *KT* kidney transplantation, *ATG* anti-thymocyte globulin

### Valacyclovir-based prophylaxis status and side effects

Table 2 summarizes the prophylaxis duration, drug dosage, and side effects observed in the valacyclovir prophylaxis group (*n* = 291) after matching. The mean duration of valacyclovir prophylaxis was 14.68 weeks and the mean dosage of valacyclovir was 2639.46 mg/day. The most common side effect was hematologic complications (12.0%) including neutropenia, thrombocytopenia, and anemia. Other adverse events included renal dysfunction in 5 patients (1.7%), gastrointestinal symptoms in 4 patients (1.4%), and other unspecified side effects in 1 patient (0.3%). No neurologic symptoms were reported. These findings provide insights into the safety profile of valacyclovir in this study cohort.

**Table 2:** Valacyclovir prophylaxis status and side effects

Variable	After matching (*n* = 291)
Duration of prophylaxis (weeks)	14.68 ± 10.08*
Drug dosage (mg/day)	2639.46 ± 2163.36
Side effects	
Hematologic complication	35 (12.0)
Renal dysfunction	5 (1.7)
Gastrointestinal symptoms	4 (1.4)
Neurologic symptoms	0
Others	1 (0.3)

^*^minimum 12 weeks and maximum 77 weeks

### Clinical outcomes according to valacyclovir prophylaxis

**Fig. 2:**
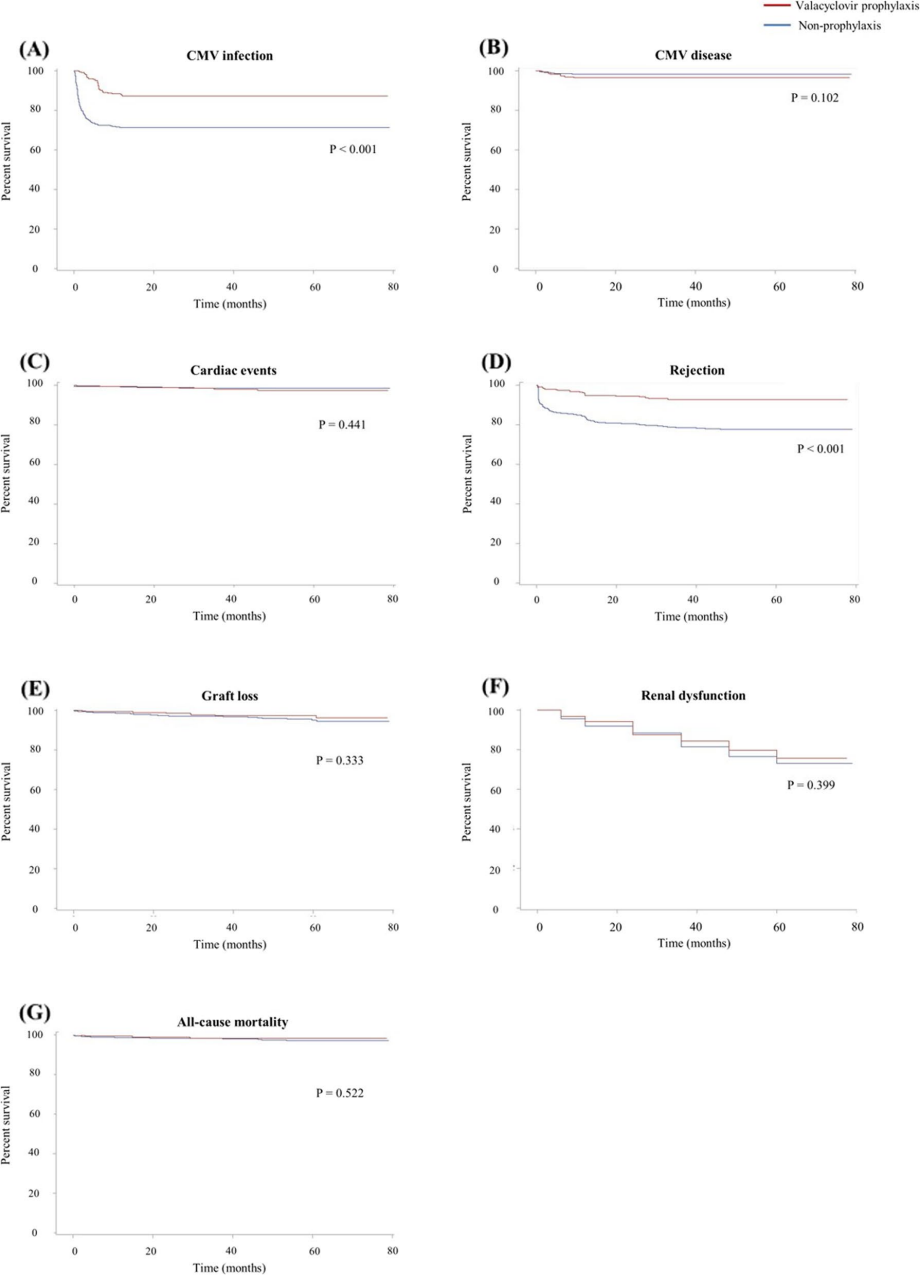
Clinical outcomes according to valacyclovir prophylaxis. CMV; cytomegalovirus

The primary and secondary clinical outcomes according to valacyclovir prophylaxis are summarized in Table 3 and Fig. 2. Within one year after KT, 251 (24.2%) and 23 (2.2%) recipients experienced CMV infection and CMV disease, respectively. Compared to the non-prophylaxis group, the prophylaxis group experienced less CMV infection (3.64 and 10.25 per 100 person-years in the prophylaxis and non-prophylaxis groups, respectively) and showed a significantly lower risk of CMV infection (HR 0.38, 95% CI 0.27–0.53, *p* < 0.01). There was no significant difference between the two groups in the occurrence of CMV disease. During the mean follow-up period of 43.5 months, 16 (1.5%) recipients experienced cardiac events, 176 (17.0%) experienced rejection, 36 (3.5%) experienced graft loss, 189 (18.2%) experienced renal dysfunction, and 22 (2.1%) experienced all-cause mortality. Graft rejection events were significantly lower in the prophylaxis group (1.85 events per 100 person-years) compared to the non-prophylaxis group (7.27 events per 100 person-years), with an HR of 0.29 (95% CI: 0.19–0.46, *p* < 0.01). However, valacyclovir prophylaxis did not significantly affect the occurrence and risk of cardiac events, graft loss, renal dysfunction, or all-cause mortality.

**Table 3:** Clinical outcomes according to CMV prophylaxis

	Prophylaxis (*n* = 291)		Non-prophylaxis (*n* = 745)			
	No. of events	No. of events/100 person-years	No. of events	No. of events/100 person-years	HR (95% CI)	*p*
CMV infection	37	3.64	214	10.25	0.38 (0.27–0.53)	< 0.01
CMV disease	10	0.91	13	0.48	1.95 (0.86–4.43)	0.11
Pneumonia	0	0	4	0.15		
Gastrointestinal disease	10	0.91	3	0.11		
Encephalitis and ventriculitis	0	0	1	0.04		
CMV syndrome	0	0	2	0.07		
Cardiac events	6	0.53	10	0.37	1.48 (0.54–4.05)	0.44
Rejection	20	1.85	156	7.27	0.29 (0.19–0.46)	< 0.01
Graft loss	8	0.71	28	1.05	0.68 (0.31–1.49)	0.34
Renal dysfunction	51	4.95	138	5.69	0.87 (0.64–1.20)	0.40
All-cause mortality	5	0.44	17	0.63	0.72 (0.27–1.95)	0.52

*CMV* cytomegalovirus, *HR* hazard ratio, *CI* confidence interval

### Risk factors for CMV infection

Univariate and multivariate Cox regression analyses were conducted to identify predictors of CMV infection after KT (Table 4). In univariate Cox regression analysis, valacyclovir prophylaxis, duration of dialysis, creatinine level at discharge, albumin level, donor age, whether the donor was deceased or not, length of hospitalization after KT, ATG usage, and serological mismatch between the donor and the recipient (CMV seropositive donor and CMV seronegative recipient) showed a significant association with the occurrence of CMV infection within one year after KT. After adjusting for variables with a *p*-value < 0.10 in the univariate analysis or variables reported to be clinically relevant, valacyclovir prophylaxis (HR 0.25, 95% CI 0.17–0.36, *p* < 0.01), donor age (HR 1.02, 95% CI 1.0–1.03, *p* = 0.01), deceased donor (HR 181, 95% CI 1.26–262, *p* < 0.01), length of hospitalization after KT (HR 1.03, 95% CI 1.01–1.04, *p* < 0.01), ATG usage (HR 3.78, 95%CI 2.67–5.33, *p* < 0.01), and serological mismatch between the donor and the recipient (HR 3.23, 95%CI 1.51–2.72, *p* < 0.01) were independent factors associated with CMV infection occurrence after KT.

**Table 4:** Predictors of CMV infection in univariate and multivariate Cox regression analyses

Variable	Univariate			Multivariate		
	HR	95% CI	*p*	HR	95% CI	*p*
CMV prophylaxis	0.49	(0.38- 0.65)	< 0.01	0.38	(0.29- 0.51)	< 0.01
Age (years)	0.02	(1.01- 1)	< 0.01	1.01	(0.99- 1.02)	0.29
Sex- male (%)	1.01	(0.77- 1.33)	0.92	1.05	(0.78- 1.42)	0.75
Cause of ESRD						
Diabetes mellitus	0.6	(0.37- 0.96)	0.03			
Hypertension	1.25	(0.78- 2)	0.36			
Glomerulonephritis	0.6	(0.39- 0.92)	0.02			
ADPKD	1.02	(0.53- 1.97)	0.96			
Others	0.99	(0.53- 1.87)	0.98			
Unknown	Ref (1)					
Duration of dialysis (months)	1.01	(1- 1.01)	< 0.01	1.00	(1- 1.01)	0.12
Diabetes mellitus	0.73	(0.54- 1)	0.05	0.80	(0.57- 1.12)	0.2
Hypertension	1.04	(0.69- 1.58)	0.85			
Cardiovascular disease	1.39	(0.94- 2.08)	0.10			
Albumin (g/dL)	1.27	(1- 1.62)	0.05	1.16	(0.92- 1.46)	0.21
Hemoglobin (g/dL)	1.05	(0.96- 1.14)	0.29			
Creatinine- baseline (mg/dL)	1	(0.96- 1.04)	0.91			
Creatinine- discharge (mg/dL)	1.11	(0.97- 1.28)	0.13	0.94	(0.78- 1.13)	0.49
Re-transplantation	1.08	(0.69- 1.68)	0.75			
Donor age (years)	1.01	(1- 1.03)	0.03	1.02	(1- 1.03)	0.01
Donor sex- male	1.12	(0.85- 1.46)	0.42			
Deceased donor	2.6	(1.99- 3.4)	< 0.01	1.56	(1.03–2.37)	0.04
Desensitization	0.81	(0.58- 1.13)	0.21	1.18	(0.69–2.03)	0.54
IVIG	0.62	(0.33- 1.14)	0.12			
Plasmapheresis	1.02	(0.67- 1.56)	0.92			
Rituximab	0.94	(0.6- 1.48)	0.80			
ABO incompatibility	1.01	(0.65- 1.57)	0.97	1.00	(0.5- 1.98)	0.99
Presence of DSA	1.18	(0.69- 2.03)	0.54	0.69	(0.38–1.26)	0.23
Length of hospitalization after KT (days)	1.02	(1.01- 1.04)	< 0.01	1.02	(1.01–1.03)	0.03
Induction immunosuppression						
Basiliximab	Ref (1)			Ref (1)		
ATG	3.46	(2.64- 4.53)	< 0.01	3.50	(2.5–4.9)	< 0.01
Mycophenolate	0.97	(0.54–1.74)	0.92	0.93	(0.47–1.83)	0.83
Steroid	2.57	(0.36–18.26)	0.35	2.48	(0.32–19.52)	0.39
Donor CMV IgG + and Recipient CMV IgG −	2.02	(1.22–3.32)	0.01	3.09	(1.77- 5.4)	< 0.01

*CMV* cytomegalovirus, *HR* hazard ratio, *CI* confidence interval, *ESRD* end-stage renal disease, *ADPKD* autosomal dominant polycystic kidney disease, *IVIG* intravenous immunoglobulin, *DSA* donor specific antibody, *KT* kidney transplantation, *ATG* anti-thymocyte globulin

## Discussion

In the present study, we analyzed the clinical efficacy of oral valacyclovir-based CMV prophylaxis 3 months in KT recipients and identified independent factors associated with the development of CMV infection using a well-organized nationwide KT cohort database. Our principal findings are as follows; First, the valacyclovir prophylaxis group experienced significantly less CMV infection and had a lower risk of CMV infection than the non-prophylaxis group without significant adverse effects. Second, valacyclovir-based prophylaxis significantly reduced the incidence and risk of graft rejection. Third, valacyclovir prophylaxis, donor age, deceased donor, length of hospitalization after KT, ATG usage, and CMV serological mismatch between the donor and recipient (CMV serological status: D + /R −) were independently associated with the development of CMV infection.

Primary CMV infection generally occurs in childhood, most of which are asymptomatic, and some patients experience mononucleosis syndrome. Seroprevalence in adults varies from country to country and is reported to be 45–100% [27]. After the primary infection, the virus remains latent in myeloid and lymphoid cells, and under conditions of immunosuppression such as acquired immunodeficiency syndrome and immunosuppressant use, this latent virus is reactivated and may contribute to chronic disease and multiorgan disorders [28–30].

The risk of opportunistic infection increases in transplant recipients who are severely immunocompromised, and CMV is a leading cause of infectious complications in the early period of KT [31]. CMV infection after organ transplantation can develop due to the reactivation of latent infection, transmission from transplanted organs, and primary infection in seronegative recipients [32, 33]. It has been reported that CMV has indirect effects on KT recipients, including increased risk of other infections, graft rejection and mortality [33]. Therefore, prevention of CMV is considered essential after KT, and international guidelines recommend the administration of ganciclovir or valganciclovir to all recipients at risk for CMV infection (CMV serological status: D + /R − , D + /R + , or D − /R +), starting within 10 days after transplantation and continuing for 3–6 months [16–18]. Valganciclovir is the preferred anti-viral agent for CMV prevention; however in actual clinical practice, it cannot be used in all recipients who require prevention owing to its limitations such as high price, economic burden, and side effects [34, 35]. In Korea, the high cost of valganciclovir, which is not covered by the national health insurance except for high-risk patients, poses a significant financial barrier. As a result, many patients receive alternative approaches, such as preemptive therapy involving regular CMV PCR monitoring instead of universal prophylaxis. These challenges are reflected in our study cohort, where a substantial number of patients in the non-prophylaxis group did not receive anti-viral agents for CMV prophylaxis, likely due to these economic and coverage limitations. This financial constraint can influence the choice of treatment strategy and impact clinical outcomes, emphasizing the need for cost-effective and widely accessible CMV prevention strategies in real-world settings.

Some researchers have suggested valacyclovir, a pro-drug of acyclovir with higher bioavailability, as an alternative to CMV prophylaxis after organ transplantation. Valacyclovir has been documented to be an effective strategy to prevent CMV after organ transplantation with less bone marrow suppression and at relatively low cost [20, 36, 37]. In this study, we observed that valacyclovir-based prophylaxis significantly reduced the incidence and risk of CMV infection after KT. Similar to prior studies, among the prophylaxis group, 37 (12.7%) recipients experienced CMV infection [20, 38]. The incidence rates of CMV infection after KT in the prophylaxis and non-prophylaxis groups were 3.64 and 10.25 events per 100 person-years, respectively. On the other hand, we did not observe an effect of valacyclovir in reducing the incidence of CMV disease. We did not observe a significant effect of valacyclovir in reducing CMV disease incidence, potentially due to the low occurrence rate, which limited statistical power. The relatively lower observation of CMV disease compared to CMV infection is presumed to be due to the widespread use of preemptive therapy in Korea, stemming from various limitations associated with CMV prophylaxis. Additionally, CMV disease diagnosis often requires active diagnostic procedures such as histological confirmation, typically employed by centers using proactive prophylaxis strategies. This may have contributed to the higher observed incidence in the prophylaxis group. The limitations inherent in our study design and data availability precluded detailed subgroup analyses on this aspect. Future research should address these limitations to provide more definitive insights.

Consistent with prior studies, the valacyclovir prophylaxis group showed a significantly lower incidence and risk of graft rejection than the non-prophylaxis group in our study. Park et al. [39] reported that KT recipients receiving valacyclovir prophylaxis for 3 months experienced lower acute allograft rejection than recipients who received intravenous ganciclovir prophylaxis for 2 weeks to prevent CMV. A recent network meta-analysis demonstrated that valacyclovir had the lowest tendency for acute rejection among different antiviral agents in patients with solid organ transplantation [40]. Considering that rejection has a major impact on recipient and graft survival, reduction in the risk of rejection through CMV prophylaxis has clinical significance in KT recipients. The mechanisms by which valacyclovir reduces rejection have not been extensively studied, and the exact molecular mechanisms remain unclear. Several possible explanations can be considered. First, valacyclovir may reduce the risk of rejection through its antiviral activity against CMV. Previous studies have demonstrated that CMV infection increases the risk of rejection following transplantation, and therefore, CMV prevention with valacyclovir could contribute to mitigating this risk. Additionally, valacyclovir may possess immunomodulatory properties beyond its antiviral effects [41], which might help reduce the likelihood of rejection. However, these observations are primarily based on laboratory studies. Further research is necessary to elucidate the potential anti-inflammatory and immunomodulatory effects of valacyclovir in clinical settings.

In our study, the baseline characteristics of the prophylaxis group revealed higher frequencies of re-transplantation and the presence of DSA compared to the non-prophylaxis group. We hypothesize that patients undergoing re-transplantation or those with DSA are more likely to receive desensitization, which may lead clinicians to be more vigilant about CMV risk and subsequently implement prophylaxis. The finding that valacyclovir prophylaxis reduced the incidence of CMV infection and rejection in this high-risk population can be regarded as significant.

The risk of CMV after KT depends on various factors, including CMV serological status of the recipient and donor, the net state of immunosuppression, and other viral and host factors [6, 7]. In our study, prophylaxis with valacyclovir, donor age, deceased donor, length of hospitalization after KT, ATG use, and CMV serological mismatch (CMV serological status: D + /R −) were independently associated with the occurrence of CMV infection. Our findings are in line with those of previous studies, and suggest that recipients with such risk factors should be considered for CMV prophylaxis.

Our study has some limitations. First, we retrospectively collected CMV-related information. Therefore, selection bias should be considered. Second, the protocol for using valacyclovir for CMV prophylaxis after KT differed for each center. The dosage of valacyclovir was not uniform, and the mean drug dosage in our study was 2639.46 mg/day, which is relatively small compared to the dosage protocol in prior studies [20, 39]. We recognize the variability in valacyclovir dosing within our cohort, reflecting differences in clinical practices across centers. Additionally, the prophylaxis duration was not consistent. While most patients had a prophylaxis duration of 12 weeks, some patients exhibited a wider range of durations. These variations may have influenced the outcomes and represent potential confounding factors in our analysis. Third, the interval for CMV monitoring after kidney transplantation was determined by each center’s protocol (Supplementary Fig. 1), which may influence the detection of CMV infection and disease. To address this limitation, future studies should be conducted prospectively with a standardized CMV monitoring schedule. Fourth, although we demonstrated the clinical efficacy of valacyclovir-based CMV prophylaxis, there is no rationale for whether valacyclovir should be considered for prophylaxis in preference to other regimens. Due to the limited number of patients receiving valganciclovir for more than three months in our cohort (*n* = 34), a comprehensive analysis was constrained. In the absence of a head-to-head comparative analysis between valacyclovir and other drugs, it is not yet known which anti-viral agent is the most appropriate for KT recipients. Larger prospective studies with standardized protocols are indeed necessary to allow for a more robust comparison between valganciclovir and valacyclovir. Fifth, the prophylaxis group had more frequent use of IVIG, but we were unable to determine its impact on providing passive immunity against CMV. It is possible that IVIG use could provide passive immunity against CMV, which might not have been fully accounted for in our study. This potential confounding factor could influence the observed outcomes and should be considered when interpreting the results. Future analyses should investigate the role of IVIG in CMV prevention to better understand its impact and whether it contributes to the differences seen between the groups. Sixth, we did not assess hematologic complications in the non-prophylaxis group. Since hematologic complications can also result from the immunosuppressive agents used by KT patients, it is necessary to compare the differences between the prophylaxis and non-prophylaxis groups. Seventh, we did not categorize rejection types or analyze the timing of rejection events relative to valacyclovir use. Additionally, data on whether prophylaxis strategies were modified following rejection were unavailable. Furthermore, our study did not include data on whether patients who developed rejection in the first year after transplantation received additional CMV prophylaxis following rejection treatment. These limitations should be addressed in future studies by collecting detailed information on rejection types, timing, prophylaxis adjustments, and post-rejection prophylaxis to provide a more comprehensive understanding. Eighth, our study identified ATG and CMV D + /R − as significant risk factors for CMV infection but did not clarify their relationship with valacyclovir prophylaxis. A detailed subgroup analysis is needed to better understand the interactions between ATG, CMV D + /R − , valacyclovir, and CMV infection in high-risk patients. Ninth, our study could not assess tacrolimus levels, which could influence outcomes and represent a potential confounding factor. Additionally, data on absolute lymphocyte counts, which may relate to viral-specific T-cell responses, were not available. Future studies should include detailed tacrolimus level data and immune profiling, including lymphocyte counts, to address these limitations comprehensively. Tenth, our cohort included patients across high, intermediate, and low risk for CMV infection to provide a comprehensive representation of real-world clinical practices. However, prioritizing high-risk groups, such as CMV D + /R − patients, is essential in accordance with current recommendations. A subset of high-risk patients (*n* = 24) did not receive prophylaxis due to non-compliance or clinician decisions. While analyzing this group to determine whether the lack of prophylaxis leads to greater harm would provide valuable insights, the small sample size and dataset limitations prevented such analysis. Future studies with larger, standardized cohorts are needed to provide targeted insights and align with guidelines. Finally, the KT recipients analyzed in the present study were predominantly Korean. Considering that trends in CMV infection and disease may differ according to ethnicity and socioeconomic status [42], our findings should be generalized with caution.

## Conclusions

In conclusion, oral valacyclovir-based prophylaxis for three months after KT significantly reduced CMV infection and graft rejection. Our findings suggest that valacyclovir could be considered as an alternative to conventional strategies for CMV prophylaxis after KT. However, our study has several limitations, including its retrospective design, variability in valacyclovir dosing and CMV monitoring protocols across centers, and a limited number of high-risk patients receiving valganciclovir. Additionally, potential confounding factors, such as IVIG use, tacrolimus levels, and rejection timing, were not fully assessed. These limitations may have influenced the outcomes, and caution is needed when interpreting our findings. Future prospective studies with standardized protocols and larger, more diverse cohorts are essential to validate these results.

## Supplementary Information

Supplementary Material 1.

## Data Availability

The datasets used and/or analysed during the current study available from the corresponding author on reasonable request.

## Abbreviations

ATG: Anti-thymocyte globulin
CI: Confidence intervals
CKD-EPI: Chronic Kidney Disease Epidemiology Collaboration
CMV: Cytomegalovirus
D: Donor
DSA: Donor-specific antibody
eGFR: Estimated glomerular filtration rate
ESRD: End stage renal disease
HRs: Hazard ratios
IVIG: Intravenous immunoglobulin
KOTRY: Korean Organ Transplantation Registry
KT: Kidney transplantation
PCR: Polymerase chain reaction
R: Recipient
SDs: Standard deviations
