# Supplementary material for: Valacyclovir for the prevention of cytomegalovirus infection after kidney transplantation
Source: BMC Infect Dis. 2025 Mar 5;25:314. doi: 10.1186/s12879-025-10671-6 (PMC11881300; doi:10.1186/s12879-025-10671-6)
Supplement: Supplementary file 1 — Supplementary Material 1. [file 12879_2025_10671_MOESM1_ESM.zip › Supplementary figure legend.docx]

**Supplementary figure legend**

**Supplementary Figure 1. CMV monitoring schedules during the first year post-transplantation.** Data were obtained through a survey from 15 of the 20 kidney transplant centers participating in the study.
