# Supplementary figures and images for: Valacyclovir for the prevention of cytomegalovirus infection after kidney transplantation
Source: BMC Infect Dis. 2025 Mar 5;25:314. doi: 10.1186/s12879-025-10671-6 (PMC11881300; doi:10.1186/s12879-025-10671-6)

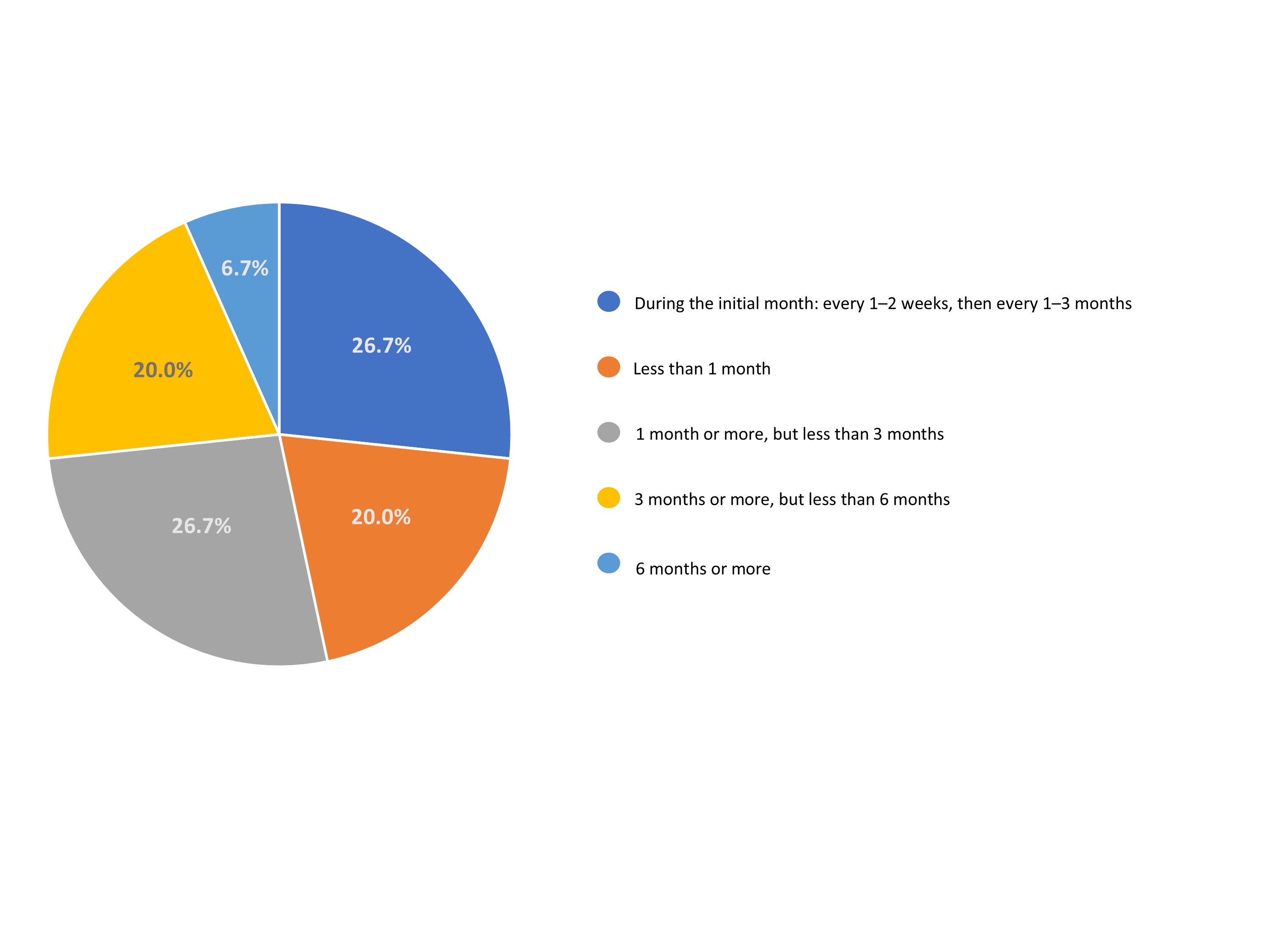

Supplement: Supplementary file 1 — Supplementary Material 1. [file 12879_2025_10671_MOESM1_ESM.zip › Supplementary Figure 1.jpg]
